# Supplementary material for: Male bonobo mating strategies target female fertile windows despite noisy ovulatory signals during sexual swelling
Source: PLoS Biol. 2025 Dec 9;23(12):e3003503. doi: 10.1371/journal.pbio.3003503 (PMC12688130; doi:10.1371/journal.pbio.3003503)
Supplement: S1 Fig — This figure presents significant variables in GLMM-1C. The subfigures show the difference in the (A) infant age of females, (B) days from the onset of maximal swelling phase (MSP), and (C) age of females depending on the existence of male IFB (O) or not (X). Although GLMM-1C indicated that males followed older females more, this result might be erroneous given the distribution of female age as shown in (C). The upper and lower edges of the box represent the 75th and 25th percentiles, respectively. A line within a box is the median, and whiskers are 1.5 times the interquartile range, and colored markers are all data points of the 9 subject females. The data and R code underlying this figure can be found in the Figshare repository (https://doi.org/10.6084/m9.figshare.30403564, https://doi.org/10.6084/m9.figshare.30405073). (PDF) [file pbio.3003503.s001.pdf]

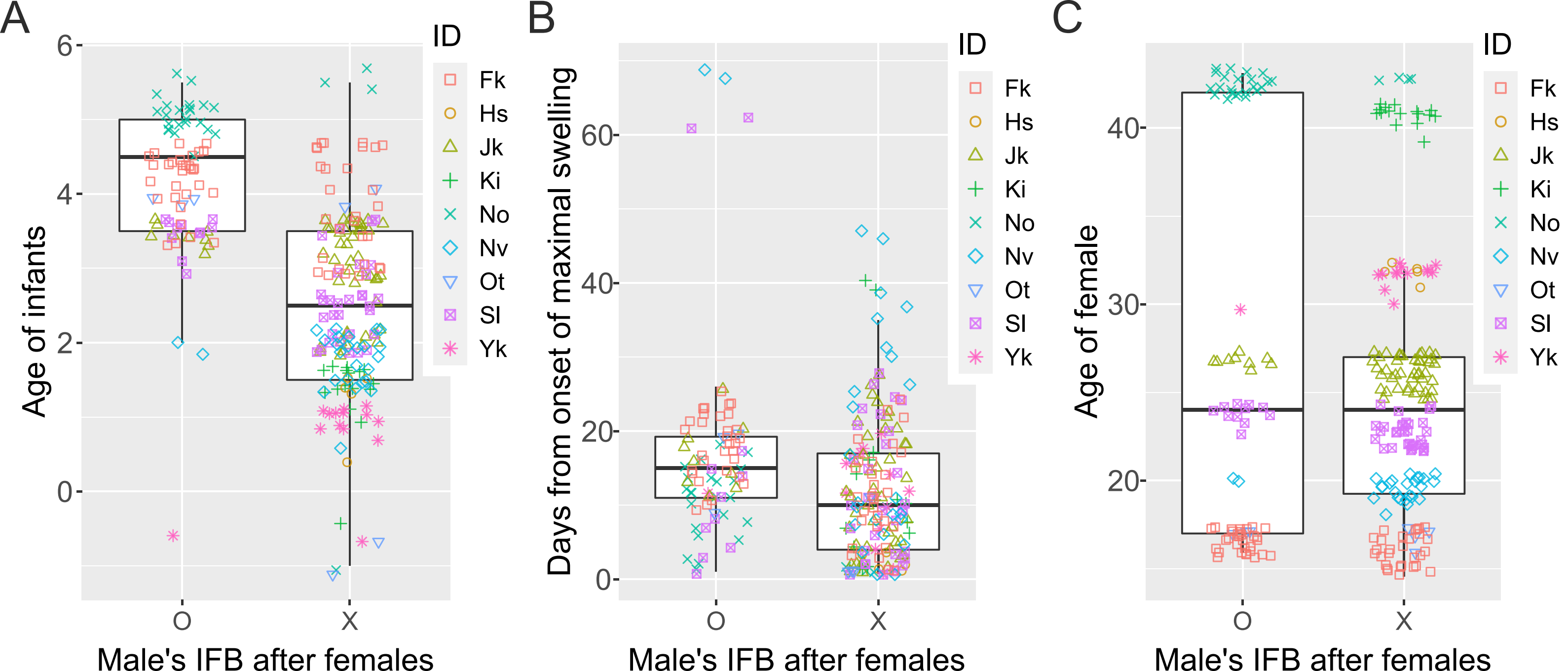

A
Age of infants
6
4
2
0
O
X
Male's IFB after females
ID
Fk
Hs
Jk
Ki
No
Nv
Ot
Sl
Yk
B
Days from onset of maximal swelling
60
40
20
0
O
X
Male's IFB after females
ID
Fk
Hs
Jk
Ki
No
Nv
Ot
Sl
Yk
C
Age of female
40
30
20
O
X
Male's IFB after females
ID
Fk
Hs
Jk
Ki
No
Nv
Ot
Sl
Yk
