# Supplementary figures and images for: Male bonobo mating strategies target female fertile windows despite noisy ovulatory signals during sexual swelling
Source: PLoS Biol. 2025 Dec 9;23(12):e3003503. doi: 10.1371/journal.pbio.3003503 (PMC12688130; doi:10.1371/journal.pbio.3003503)

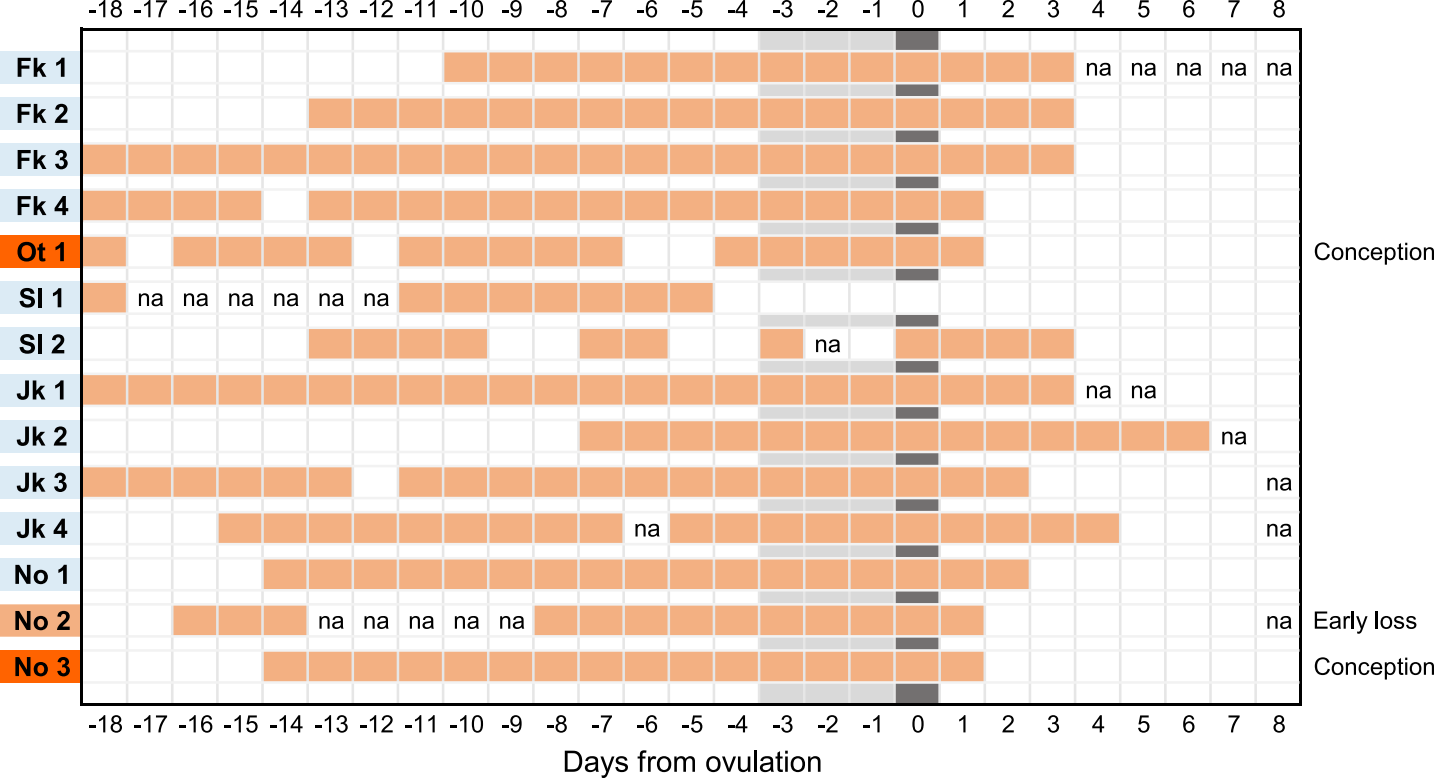

Supplement: S2 Fig — A fertile phase (periovulatory phase) is defined from −3 to 0 days from ovulation. The onset of the MSP is the first day maximal swelling was observed. For Sl 1, the length (days) of MSP was defined from −18 to 0 days from ovulation. When the swelling score temporarily dropped to 2 (intermediate swelling) from 3 (maximal swelling), then rose again to 3 within 4 days, we considered MSP was continuous. The orange block represents the day that females were in the MSP (swelling 3), the white block represents not maximal swelling (swelling 1 or 2), na: absent of the female. https://doi.org/10.6084/m9.figshare.30405127. (PDF) [file pbio.3003503.s002.pdf]

Male rank — High - - Low

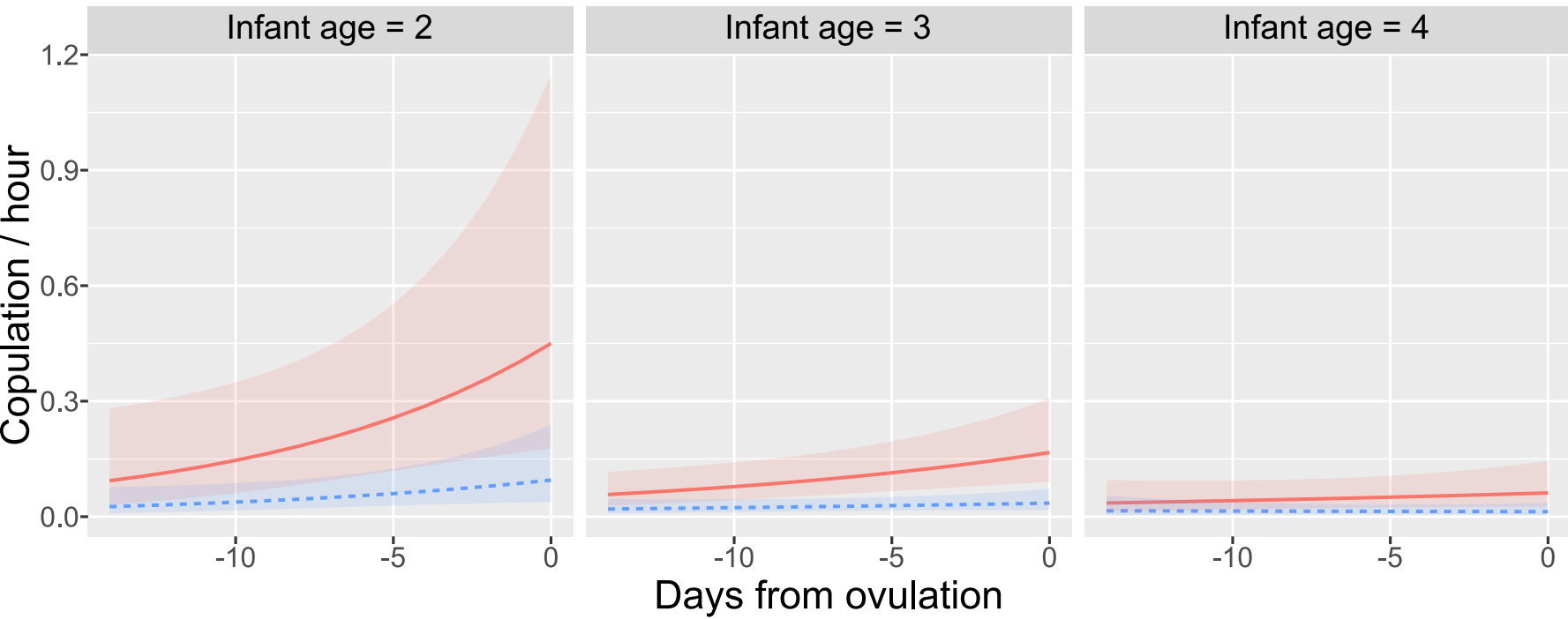

Male rank — High - - Low

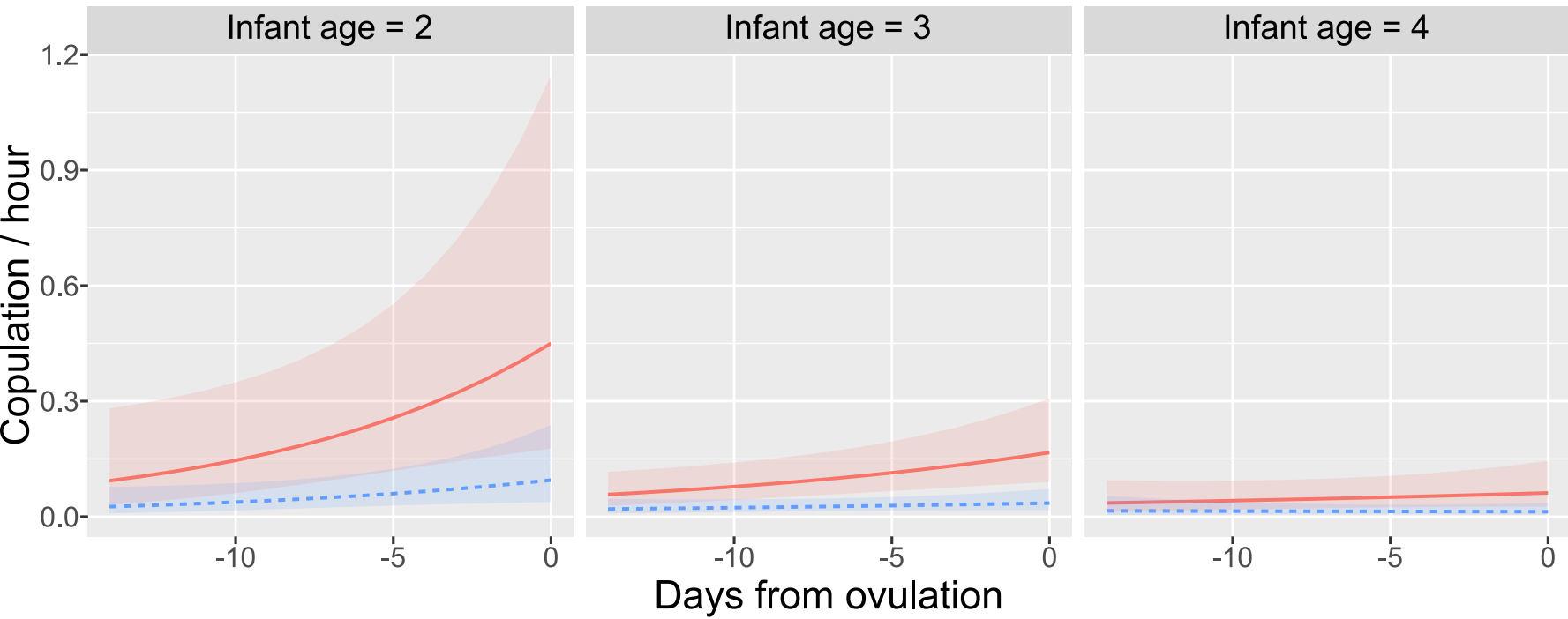

Supplement: S3 Fig — This figure presents the significant 3-way interaction term demonstrating that males copulated more with females with younger infants than those with older infants. Such a tendency was clearer for the three high-ranking males. Colored bands (ribbon) around each fitted line represent the 95% confidence interval (CI) from GLMM-2B. The data and R code underlying this figure can be found in the Figshare repository (https://doi.org/10.6084/m9.figshare.30403564, https://doi.org/10.6084/m9.figshare.30405190). (PDF) [file pbio.3003503.s003.pdf]

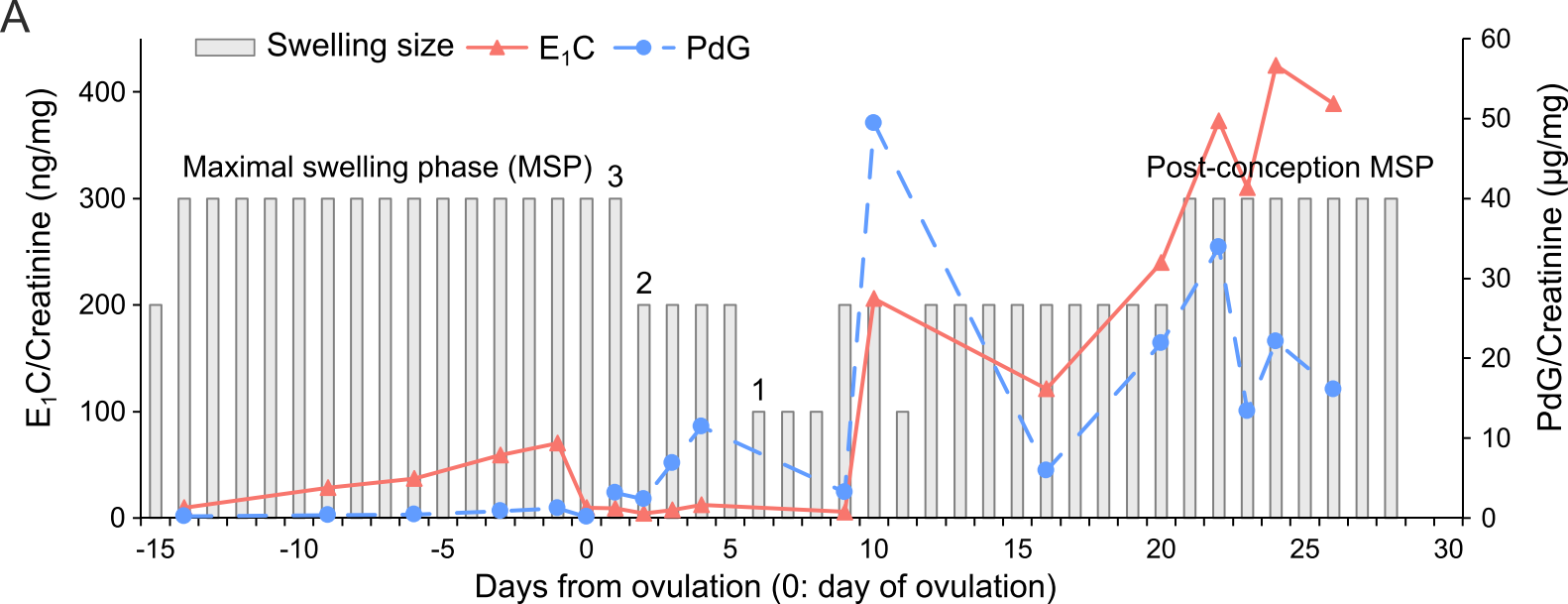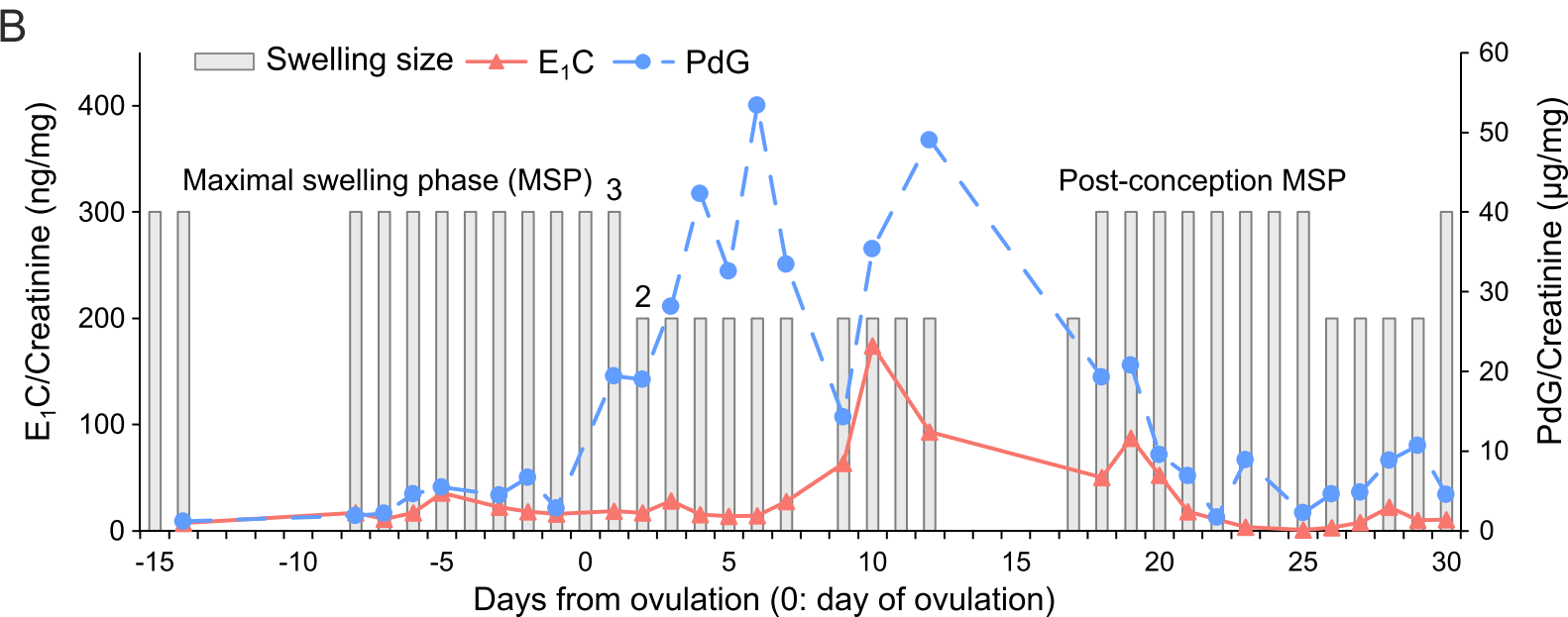

Supplement: S4 Fig — (A) A conceptive menstrual cycle of a female, No, that resulted in a successful delivery. An earlier surge of estrogen (E1C) than progesterone (PdG) resulted in postconception maximal swelling phase (MSP) after 3 weeks from ovulation. (B) A conceptive but miscarriage occurred menstrual cycle of the same female, No. Although there was a surge of estrogen and progesterone, after around 10 days from ovulation, this cycle failed in keeping a high concentration of estrogen and progesterone, so an early loss of pregnancy occurred. It is notable, however, that a short MSP came as in the successful conceptive cycle shown in (A). The data and R code underlying this figure can be found in the Figshare repository (https://doi.org/10.6084/m9.figshare.30403564, https://doi.org/10.6084/m9.figshare.30405220). (PDF) [file pbio.3003503.s004.pdf]

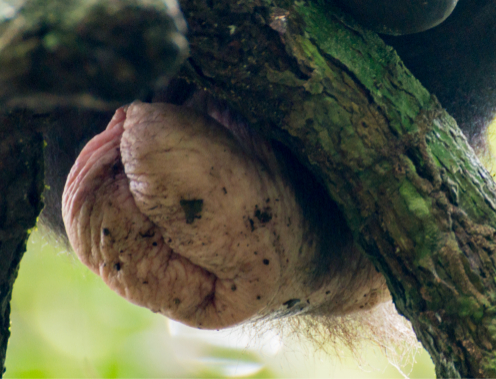

Non swelling (score 1)

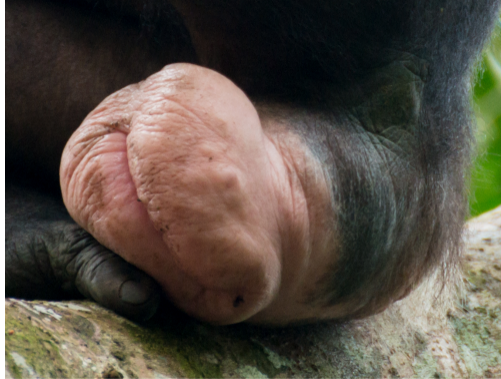

Intermediate swelling (score 2)

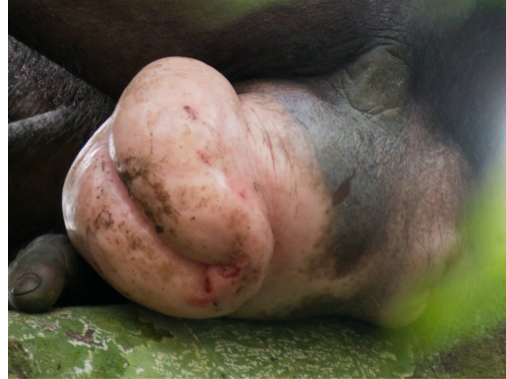

Maximal swelling (score 3)

Supplement: S5 Fig — Morphological changes in sexual swelling of a female, Fk. Non-swelling status was scored 1, intermediate swelling was scored 2, and the maximal swelling was scored 3. https://doi.org/10.6084/m9.figshare.30405235. (PDF) [file pbio.3003503.s005.pdf]
