# Supplementary material for: Male bonobo mating strategies target female fertile windows despite noisy ovulatory signals during sexual swelling
Source: PLoS Biol. 2025 Dec 9;23(12):e3003503. doi: 10.1371/journal.pbio.3003503 (PMC12688130; doi:10.1371/journal.pbio.3003503)
Supplement: S1 Table — The age of each female in 2015 was estimated based on immigration date and several morphological cues, while the age of males was estimated mostly based on their birth date, with morphological cues. The immigration dates of Yk, Hs, Jk, and Sl were the year and month when they were identified. The infant age in April 2015 is expressed in months. Ovulation cycles: ovulation detected menstrual cycles, (anovulation): anovulatory cycle. maximal swelling phases (MSP): The number of MSPs defined for the female. †, ‡: infant births within 2 weeks. https://doi.org/10.6084/m9.figshare.30405247. (PDF) [file pbio.3003503.s006.pdf]

**S1 Table. Basic information about subject individuals in E1 group.**

**Females and their youngest infants**

| Name | Age (2015) | Infant age (2015.04) | Ovulation cycles (anovulation) | MSP | Urine samples | Immigration | Latest delivery |
|---|---|---|---|---|---|---|---|
| No | 44 | 0 | 3 (0) | 9 | 120 | 1983.11 | †2015.04 |
| Ki | 41 | 15 | 0 (0) | 4 | 44 | 1984.12 | ‡2014.02 |
| Yk | 34 | 13 | 0 (0) | 5 | 29 | *2004.04 | 2014.04 |
| Hs | 32 | 15 | 0 (2) | 4 | 36 | *2003.08 | ‡2014.02 |
| Jk | 27 | 39 | 4 (0) | 7 | 60 | *2004.04 | 2012.01 |
| Sl | 24 | 40 | 2 (0) | 9 | 114 | *2003.08 | 2011.12 |
| Nv | 20 | 21 | 0 (2) | 5 | 53 | 2007.08 | 2013.7 |
| Ot | 18 | 0 | 1 (0) | 3 | 65 | 2008.06 | †2015.04 |
| Fk | 17 | 51 | 4 (1) | 7 | 139 | 2008.06 | 2011.02 |

**Males**

| Name | Age (2015) | Age class | Copulation | Rank SP1 | Rank SP2&3 | Identified (age) |
|---|---|---|---|---|---|---|
| TN | 45 | Old | 21 | 8 | 8 | 1976 (6) |
| TW | 41 | Old | 0 | 7 | 7 | 1976 (2) |
| DI | 40 | Old | 20 | 9 | 10 | 2004 (29) |
| NB | 27 | Middle | 68 | 2 | 2 | 1988 (0) |
| GC | 27 | Middle | 18 | 4 | 3 | 2003 (15) |
| LB | 22 | Middle | 18 | 5 | 6 | 2003 (12) |
| JD | 22 | Middle | 39 | 6 | 4 | 2003 (12) |
| JR | 14 | Young | 34 | 1 | 5 | 2004 (3) |
| KT | 11 | Young | 274 | 3 | 1 | 2004 (0) |
| SB | 11 | Young | 78 | 10 | 9 | 2004 |
