# Supplementary material for: Male bonobo mating strategies target female fertile windows despite noisy ovulatory signals during sexual swelling
Source: PLoS Biol. 2025 Dec 9;23(12):e3003503. doi: 10.1371/journal.pbio.3003503 (PMC12688130; doi:10.1371/journal.pbio.3003503)
Supplement: S2 Table — In study period 1 (SP1), JR occupied the highest rank. However, the h′ index (0.84) indicated that the hierarchy was not linear. In SP2 & 3, the hierarchy was linear (h′ = 0.95). The numbers next to the name represent the age of the individual in 2014 (SP1) and 2015 (SP2 & 3). https://doi.org/10.6084/m9.figshare.30405262. (PDF) [file pbio.3003503.s007.pdf]

S2 Table. The number of male dyadic agonistic interactions during the study periods.

SP1

ID	JR	NB	KT	GC	LB	JD	TW	TN	DI	SB
JR (13)		10	13	21	9	10	1	5	2	20
NB (26)	6		0	30	4	9	0	5	1	12
KT (10)	0	5		2	8	4	0	8	2	15
GC (26)	4	2	1		10	0	2	4	1	2
LB (20)	0	0	2	0		1	5	3	7	6
JD (20)	0	0	0	0	0		3	2	7	18
TW (40)	0	0	0	1	0	0		3	0	0
TN (44)	0	0	0	0	1	0	0		8	8
DI (39)	0	0	1	0	0	0	1	0		9
SB (10)	0	0	0	0	0	0	0	0	0	

SP2&3

ID	KT	NB	GC	JD	JR	LB	TW	TN	SB	DI
KT (11)		40	15	6	20	26	1	3	28	4
NB (27)	2		31	8	56	19	3	7	8	4
GC (27)	0	0		1	84	6	2	4	10	9
JD (21)	0	0	0		0	11	5	5	9	9
JR (14)	3	8	0	0		1	0	1	2	2
LB (21)	0	0	0	0	0		8	7	4	25
TW (41)	0	0	0	0	0	0		2	0	1
TN (45)	0	0	0	0	0	0	0		1	8
SB (11)	2	0	0	0	0	0	0	0		0
DI (40)	0	0	0	0	0	0	0	0	3	
