# Supplementary material for: Male bonobo mating strategies target female fertile windows despite noisy ovulatory signals during sexual swelling
Source: PLoS Biol. 2025 Dec 9;23(12):e3003503. doi: 10.1371/journal.pbio.3003503 (PMC12688130; doi:10.1371/journal.pbio.3003503)
Supplement: Attachment — Submitted filename: Response to Reviewers.docx [file pbio.3003503.s011.docx]

Dear Dr Ryu,

Thank you for your patience while your manuscript "Sexual swelling in bonobos: variations, ovulation predictability, and male responses" was peer-reviewed at PLOS Biology. It has now been evaluated by the PLOS Biology editors, an Academic Editor with relevant expertise, and by several independent reviewers.

In light of the reviews, which you will find at the end of this email, we would like to invite you to revise the work to thoroughly address the reviewers' reports. As you will see, the reviewers describe the study as well carried-out and the results as interesting. However, they all expressed concerns about the narrow focus of the study on bonobos and their differences from chimpanzees. They also all pointed out organizational and clarity issues that impeded following the results, and some additional concerns over controls, statistics, and methodological details.

Your revision will need to carefully consider and address these points. In accordance with our next point, we think that adding direct bonobo/chimpanzee comparisons is not critical here, but you should address all other concerns raised.

IMPORTANT: Crucially, for your study to be within scope for publication in PLOS Biology, you will need to substantially broaden the Introduction and Discussion sections beyond chimpanzees and bonobos. Based on our discussion with the Academic Editor, we encourage you to contextualize your study within the broader work on male mating tactics and female signals, including those done on non-primates. See also the wording from the Academic Editor at the end of this email.

Given the extent of revision needed, we cannot make a decision about publication until we have seen the revised manuscript and your response to the reviewers' comments. Your revised manuscript is likely to be sent for further evaluation by all or a subset of the reviewers.

In addition to these revisions, you will need to complete some formatting changes, which you will receive in a follow up email. A member of our team will be in touch with a set of requests shortly.

We expect to receive your revised manuscript within 3 months. Please email us (plosbiology@plos.org) if you have any questions or concerns, or would like to request an extension.

At this stage, your manuscript remains formally under active consideration at our journal; please notify us by email if you do not intend to submit a revision so that we may withdraw it.

**IMPORTANT - SUBMITTING YOUR REVISION**

Your revisions should address the specific points made by each reviewer. Please submit the following files along with your revised manuscript:

1. A 'Response to Reviewers' file - this should detail your responses to the editorial requests, present a point-by-point response to all of the reviewers' comments, and indicate the changes made to the manuscript.

*NOTE: In your point-by-point response to the reviewers, please provide the full context of each review. Do not selectively quote paragraphs or sentences to reply to. The entire set of reviewer comments should be present in full and each specific point should be responded to individually, point by point.

You should also cite any additional relevant literature that has been published since the original submission and mention any additional citations in your response.

2. In addition to a clean copy of the manuscript, please also upload a 'track-changes' version of your manuscript that specifies the edits made. This should be uploaded as a "Revised Article with Changes Highlighted" file type.

*Re-submission Checklist*

When you are ready to resubmit your revised manuscript, please refer to this re-submission checklist: https://plos.io/Biology_Checklist

To submit a revised version of your manuscript, please go to https://www.editorialmanager.com/pbiology/ and log in as an Author. Click the link labelled 'Submissions Needing Revision' where you will find your submission record.

Please make sure to read the following important policies and guidelines while preparing your revision:

*Published Peer Review*

Please note while forming your response, if your article is accepted, you may have the opportunity to make the peer review history publicly available. The record will include editor decision letters (with reviews) and your responses to reviewer comments. If eligible, we will contact you to opt in or out. Please see here for more details:

https://blogs.plos.org/plos/2019/05/plos-journals-now-open-for-published-peer-review/

*PLOS Data Policy*

Please note that as a condition of publication PLOS' data policy (http://journals.plos.org/plosbiology/s/data-availability) requires that you make available all data used to draw the conclusions arrived at in your manuscript. If you have not already done so, you must include any data used in your manuscript either in appropriate repositories, within the body of the manuscript, or as supporting information (N.B. this includes any numerical values that were used to generate graphs, histograms etc.). For an example see here: http://www.plosbiology.org/article/info%3Adoi%2F10.1371%2Fjournal.pbio.1001908#s5

Thank you again for your submission to our journal. We hope that our editorial process has been constructive thus far, and we welcome your feedback at any time. Please don't hesitate to contact us if you have any questions or comments.

Sincerely,

Taylor

Taylor Hart, PhD,

Associate Editor

PLOS Biology

thart@plos.org

------------------------------------

Dear Dr. Taylor Hart,

Thank you for your clear guidance and for coordinating the constructive reviews. We apologize for the significant delay in submitting our revision and hope that the revised manuscript and our responses to the reviewers meet your expectations.

In response to the editorial decision letter and reviewer comments, we have revised the manuscript to address key concerns regarding scope, organization, and clarity. Specifically, we rewrote the Abstract, Introduction, and Discussion to broaden our scope beyond chimpanzees and bonobos and to situate our findings within general theory on sexual selection, male mating strategies, and female signaling across mammals. We also incorporated comparative work beyond bonobos and chimpanzees where relevant, and clarified how our results contribute to understanding the evolution and maintenance of conspicuous but probabilistic ovulatory signals in bonobos.

We also improved the manuscript’s organization and presentation of results. We reduced the use of acronyms, removed speculative statements, and re-organized the flow of the Results section. Key statistical tables previously included in the Supplementary have been moved into the main text to make numerical estimates and significance values easy to access. Figure captions have also been revised for clarity.

We consent to publication of the peer‐review history and have deposited the underlying data and R code on Figshare; the DOI is provided in the Data Availability statement.

Lastly, we appreciate the opportunity to revise and believe these changes improve the clarity, scope, and accessibility of the work while preserving its core contribution. We hope this revision meets your expectations, and we would be glad to make any further adjustments you recommend.

**Our revisions and responses to the reviewers are as follows.**

First, we would like to address an error we discovered in the coding of infant age data used in two GLMMs (GLMM-1B and GLMM-1C). While preparing the dataset for public sharing, we identified a mislabeling in the age categories: infants aged 0–6 months were coded as 0.5, and those aged 6–12 months were coded as 1.0, and so on. We corrected the dataset for the two GLMMs so that age 0 now represents 0–6 months, age 0.5 represents 6–12 months, and so forth. All other models in the manuscript used correctly coded infant ages and remain unchanged. Importantly, this correction did not affect the statistical significance, directionality of model fit, or reliability of the model results. Only minor numerical changes occurred in the model estimates.

Second, we have revised several sentences, especially in the Results section, to improve readability and flow. All changes are both highlighted and tracked in the manuscript.

The following are our responses to the reviewers. Our responses are guided by the right arrowhead symbol, ⮚ (Unicode Character: 2B9A).

# Response to Reviewer #1 (general comments)

**Your comments**

This is an interesting study examining what male know about female anogenital sexual swellings in bonobos. The study uses a strong data set to investigate the question and the methodology in general is sound. I do have some reservations detailed below however which need to be addressed:

- Thank you for your thoughtful and constructive comments on our manuscript. We greatly appreciate your recognition of the strength of our dataset and the overall soundness of our methodology. Your feedback has been instrumental in guiding substantial improvements to the revised manuscript.

In the following responses, we address each of your concerns in detail. We have revised the manuscript accordingly and hope that these changes clarify the issues you raised and meet your expectations. We are grateful for the opportunity to refine our work and believe the revisions have strengthened both the clarity and the broader relevance of our study.

**Your major points**

1. The introduction is hugely skewed towards chimp and bonobo studies. Anogenital sexual swellings are found in a wide range of primate species and much research has been conducted into this as well as overarching theories which should be detailed in the intro. This by passes a large amount of theoretical knowledge and information from other species with anogenital swellings, I would recommend re-writing the intro to focus more on the overarching theory and wide range of species which exhibit this rather than purely chimps and bonobos.

- Thank you for this insightful comment. We have substantially revised the Introduction to foreground general theory on sexual selection and female signaling, and to situate primate sexual swellings within a broader evolutionary and comparative context. We now summarize key theoretical frameworks, including the graded-signal and reliable-indicator hypotheses, and incorporate comparative evidence from a wider range of primate species such as baboons, mandrills, and macaques, including cases that challenge reliable-indicator predictions.

To reduce the ape-centric emphasis of the original draft, we restructured the text to present species-specific details within concise comparative statements. These changes help clarify the relevance of our study within the broader literature on mammalian reproductive signaling. Please see the revised Introduction (LL 73–139), where we cite representative work across taxa and explicitly state how our study builds on and contributes to this wider body of research. We hope that this revision will strengthen the theoretical foundation of the manuscript and improves its accessibility to a broader readership.

2. Discussion is very focussed on the direct results and bonobos, there is very little comparison outside of bonobos, except to chimps every so often and broadly to primates in general at the end of the discussion. I think the paper would benefit from taking a broader perspective, highlighting other studies and integrating their findings into the discussion as with in introduction.

- Thank you for your helpful suggestion and clear guidance. We have substantially revised the Discussion to reduce the ape-centric framing and broaden the comparative scope. Specifically, we removed speculative interpretations tied exclusively to bonobos and chimpanzees and reframed the discussion to explore mechanisms by which males may manage probabilistic ovulatory signals under noisy conditions. We discuss how such mechanisms could contribute to the evolution and maintenance of conspicuous fertility signals in bonobos and other mammals.

To strengthen the comparative perspective, we integrated findings from other primate species and highlighted how our results inform broader questions about sexual signaling and male mating strategies across taxa. In particular, we emphasize the role of late-phase cues as potential “stop signals” that guide male effort allocation and improve paternity estimation. This framing allows us to propose how conspicuous—but noisy—ovulatory signaling systems may evolve and persist under sexual selection.

Please see the revised Discussion (LL 354–394), where we incorporate comparative literature and clarify the broader implications of our findings. We hope these changes enhance the manuscript’s relevance and theoretical contribution.

3. None of the hypotheses focus on chimpanzees and so the whole narrative of chimp and bonobo comparison throughout the intro seems disconnected to the aim of the study, it is not a comparative study of chimps and bonobos

- In the revised manuscript, we restructured the Introduction to begin with a broader overview of the evolution of conspicuous female signaling in mammals. We then narrowed the focus to primates, emphasizing the role of visual signaling systems in sexual communication. We removed comparative narratives that previously implied a direct comparison between chimpanzees and bonobos, which were not central to our current study aims. This restructuring allowed us to clarify the scope of the study, which centers on sexual selection and the evolution of conspicuous ovulatory signaling. We also revised and simplified the statements of our aims and hypotheses (LL 150–170) to ensure they are clearly aligned with the study’s objectives. We hope these changes address your concern and improve the coherence and relevance of the Introduction.

4. Results: I think some basic numerical statistical output is necessary, if the results tables are all included as supplemental material then the estimate error and p value at least should be included in the text to demonstrate that a result is indeed significant, the reader should not have to hunt this info out.

- Thank you very much for your helpful suggestion. We fully agree with your point and have addressed it in the revision. Specifically, we moved two key results tables from the supplementary into the main manuscript. These are now presented as Tables 1 and 2, allowing readers to access numerical estimates, standard errors, and significance values directly within the Results section. We hope this change improves the clarity and accessibility of the statistical findings and eliminates the need for readers to search through supplemental materials to verify significance.

5. There is a huge body of work and several analyses in the statistics, this makes the results very dense and due to the non-reporting of numerical values it can be hard to follow, huge amounts of the results have been put into supplemental materials and so the reader must flick between the results and the supplements to understand the results. This makes it a bit disjointed. On top the figures are very dense, in essence up to 9 plots in one figure which are supposed to summarise the results rather than the actual stats. I would find it easier to follow with perhaps either some whole analyses moving to the supplements or at the very least the numerical output in the text.

- Thank you for this valuable feedback. As noted in our response to Comment 4, we have moved all essential statistical tables from the supplementary materials into the main manuscript. This includes numerical estimates and significance values, which are now directly visible to readers. We also revised and clarified figure captions to improve interpretability and ensure that readers can follow the logic of the results without needing to consult the supplement. Additionally, we relocated the comparative table on maximal swelling phases in chimpanzees and bonobos across different sites from the supplement to the main text, as it provides important context for interpreting our findings.

We acknowledge that the figures were previously dense, and while we retained the multi-panel format, we have revised the captions to better guide readers through the visual summaries. We hope these changes significantly improve the readability and accessibility of the manuscript and address your concerns regarding the presentation of statistical results.

6. It is mentioned that mother-son relationships are present in the group, how were these controlled for in the data analysis?

- Thank you for raising this important point. We carefully examined the potential influence of mother–son dyads on our results. In the dataset used for GLMMs 1A to 1D, there were no cases in which sons exhibited intensive following behavior toward their mothers. Therefore, it is unlikely that mother–son relationships biased the results of these models. If any influence had occurred, it would likely have led to an underestimation of the effect of maximal swelling, as sons typically do not follow their mothers during this phase.

This pattern also held true for other interactions, including copulation, solicitation, and intervention that were measured within male-female dyad. For the data analysis, we had already excluded mother–son dyads from GLMMs 2A to 2D, as their inclusion would have increased the number of zero values in the dataset, potentially affecting model outcomes. Importantly, ovulation was not detected in the alpha female, Ki, who is the mother of two high-ranking males (ranked 1st and 2nd). As a result, Ki did not contribute data to GLMMs 2A and 2D. Therefore, only the Jk–JR mother–son dyad was removed from the dataset in these models.

Following your suggestions, we added the following clarification to the Methods section (LL 604–605):

“The mother–son dyad (Jk–JR) was excluded from the dataset to minimize unnecessary zero inflation in the behavioral data.”

7. Line 597: why was rank categorised for this analysis but not the others? It seems more reliable to use the same rank score throughout?

- Thank you for this insightful comment. We categorized male rank only in GLMM-2B to further investigate behavioral differences between high-ranking and low-ranking males, following the results of GLMM-2A. In the field, we observed that the top three ranking males exhibited distinct behavioral patterns compared to lower-ranking males. Notably, two of the top-ranking males were brothers and often supported each other during aggressive interactions surrounding copulation, suggesting a cooperative dynamic that may not be present among lower-ranking individuals.

Additionally, using a continuous rank score in models with complex three-way interactions (e.g., ovulation probability × infant age × male rank) can be difficult to interpret and may obscure meaningful behavioral contrasts. Continuous rank scores tend to produce linear effects, which may not capture the nonlinear or threshold-based differences in behavior that we aimed to explore. By categorizing rank into high (top 3) and low (ranks 4–10), we were able to more clearly assess whether high-ranking males behave differently in response to female reproductive cues.

This approach allowed us to test whether the top-ranking males disproportionately copulated with females of varying reproductive quality (e.g., infant age), and whether their behavior was modulated by ovulation timing. We believe this categorical distinction provides a more biologically meaningful interpretation of rank-related behavioral strategies in this context.

# Response to Reviewer #1 (minor questions)

Line 86 onwards: towards the end of the intro two of the main theories relating to the function of maximal swellings are introduced, I think these should be introduced much earlier.

- We agree that introducing the two main hypotheses earlier in the introduction would improve the logical flow. However, due to substantial revisions made to the introduction, it was difficult to strictly follow your recommendation by relocating the discussion to an earlier section. Instead, we have integrated the reliable signal hypothesis and the graded signal hypothesis into the revised introduction, specifically within the broader discussion of sexual swelling in primates (see Lines 73–139). This integration allows us to present these hypotheses in the context of evolutionary and comparative perspectives on sexual signaling in mammals and primates. We hope this restructuring meets your expectations and enhances the coherence and accessibility of the theoretical framework for readers.

Line 116: only now is it mentioned that other primates have maximal swellings.

- In the revised manuscript, we have addressed this issue by introducing the occurrence of maximal swellings in other primates earlier in the introduction. Specifically, this information now appears in the section discussing female signaling in mammals and primates (see Lines 73–99). We hope this adjustment improves the clarity and logical flow of the introduction and better contextualizes the significance of sexual swelling in bonobos.

Line 141: The figure legend here is very large, perhaps consider reducing the text, often it provides an explanation for what is in the figure as well as a basic description.

- Thank you for your suggestion. We have made efforts to reduce the length of the figure legends where possible. Although following another reviewer’s recommendation, we removed abbreviations such as FMS (female with maximal swelling), MSP (maximal swelling phase), and IFB (intensive following behavior) to improve clarity for general readers, we kept those abbreviations in the figure and table legends. We hope that our revisions keep it as concise and informative as possible while maintaining clarity and accessibility.

Line 167: It is unclear what the interaction term was in this analysis.

- Following your suggestion, we revised the sentence to explicitly state the interaction term:

“a significant interaction between the day from detumescence and infant age” (Lines 185–186)

from, “a significant interaction term between the two explanatory variables”

Line 232: this sentence is unclear, please rephrase

- We revised it from

“Finally, the hourly number of aggressions between males on a given male on a day (male aggression rate) increased on the day when male IFB was observed and with an increase in the number of males (Fig 4A; LM-2E in Table B in S1 Text).”

to

“The hourly rate of male–male aggression (male aggression rate) was higher on days when male intensive following was observed and increased with the number of males present (Fig 4A; LM-2E in Table 2).” See LL 271 ~ 272

407: How many researchers collected the data? If multiple were there tests for interobserver reliability?

- All behavioral observations and urine sample collections were conducted by a single researcher – the first author.

Line 417: Instantaneous scan sample?

- We did not conduct scan sampling for all visible individuals. Instead, the scan was used to record individuals within 5 meters of the focal animal. To clarify this, we revised the sentence from:

We recorded the names and behaviors of neighboring individuals within 5m of the focal animal at 5-minute intervals, including a scan at the beginning of the focal session.

To

We recorded the names and behaviors of individuals within 5m of the focal animal at 5-minute intervals, including an initial scan at the beginning of each focal session to collect information on nearby individuals.

Line 419: as often as possible?

- Thank you for your suggestions. The phrase ‘as far as possible’ may better reflect our best efforts to collect such rare events.

Line 609: Random effects, should a random effect related to the day of study be included here and in the other models? There could be some time related effect such as fatigue of males having to mate with multiple females across a longer time period. It might also we wise to control for party size.

- Thank you for your thoughtful suggestions regarding model structure and potential confounding factors. We understand your concern about including a random effect for the day of study. However, as noted in the manuscript, the male–male aggression rate was calculated as the hourly rate per male per day, meaning the unit of analysis was already aggregated at the day level. Therefore, including “day” as a random effect was not applicable in this model.

Regarding the possibility of male fatigue influencing aggression rates, we agree this is an important consideration. If fatigue plays a role, it could help explain the observed decrease in male–male aggression when both male and female party sizes are high. While this is a very fascinating idea, we feel it remains speculative without direct behavioral or physiological data on male fatigue. We have decided to keep this possibility in mind for future research and have not included it in the current discussion to avoid overinterpretation.

As for party size, it was controlled in the model through the calculation of aggression rate. For example, if 5 males engaged in 25 aggressive interactions during 5 hours of observation on a given day, the aggression rate was calculated as 25 ÷ 5 ÷ 5 = 1 aggression/hour/male. This approach ensures that both male number and observation time are accounted for in the aggression metric.

# Response to Reviewer #2 (general comments)

**Your comments**

This ms presents new behavioral and physiological data on a community of wild bonobos that are used to explore the ability of males to detect the ovulatory window in females, who have unusually long maximal genital swellings. The authors set up several hypotheses about behavioral variation in males in response to changes in several female attributes. The results indicate that the males are able to estimate, but not to pinpoint, the probability of ovulation. The data are hard-won and valuable, the analyses appropriate, the predictions comprehensible, and the conclusions follow from the results. Yet, it is this referee's impression that this study has produced any insights that are of interest to a large biological audience. The main comparisons made when developing the equations and aims are with chimpanzees (even though there are also detailed studies of Old World monkeys), and the long Discussion also centers mainly on bonobos. While they clearly represent an enigmatic species and are closely-related to humans, I find such an ape-centric perspective problematic because it tends to limit the interest of this study to other great ape researchers. Thus, while the paper is technically sound, I do not see that the journal's criteria ("features works of exceptional significance, originality, and relevance; significant advances resulting from original lines of inquiry that have a broad impact in their field and across other disciplines") are satisfied here. Should the author's revise their ms for this or another journal, I have several queries and comments that may help to improve the ms (in their order of appearance):

- Thank you very much for your thoughtful and constructive feedback. We agree that the original framing was overly focused on bonobos, which may have limited the broader relevance of the study. In response, we have substantially revised both the Introduction (Lines 40–153) and Discussion (Lines 300–388) to better situate our findings within general questions in sexual selection, reproductive signaling, and male mating strategies across mammals. We also draw on comparative data from non-ape primates and other mammals (e.g., baboons, macaques, house mice, meerkats) to highlight convergent patterns and evolutionary trade-offs in female signaling and male response. These additions aim to clarify how our within-species analysis in bonobos informs broader questions in behavioral ecology and sexual selection.

In the Introduction, we now start by reviewing female signaling in mammals more broadly, including both olfactory and visual signals, and the evolutionary pressures shaping them. We then introduce exaggerated sexual swellings in anthropoid primates and present the reliable signal hypothesis and graded signal hypothesis within a comparative framework that includes Old World monkeys and other mammals. This revised structure clarifies how bonobos fit into a broader evolutionary continuum of female reproductive signaling.

In the Discussion, we highlight how male bonobos navigate probabilistic ovulatory signals by integrating multiple information sources, with detumescence serving as a reliable retrospective indicator of ovulation and an effective behavioral cue to cease mating effort. By timing their mating investment to detumescence patterns, males can disengage once the fertile window has closed, thereby reducing unnecessary energetic expenditure. This strategy exemplifies a broader mechanism whereby late-phase cues help stabilize signaling systems even in the presence of substantial noise. We propose that such cues – which signal fertility termination rather than initiation – may be crucial for maintaining conspicuous yet noisy and probabilistic fertility signals across species.

We hope these revisions help demonstrate the broader significance of our study and its relevance to researchers across disciplines. Thank you again for your insightful comments, which greatly improved the manuscript.

# Response to Reviewer #2 (specific comments)

**Your comments**

Line 58: it seems unlikely that the reduction in male competition represents an evolutionary benefit *for females* that could explain the length of their MSP (or is there a reference to this effect that can be cited?).

- Thank you for this important point. We acknowledge that the relevant reference was missing in the original draft. The idea originates from Furuichi (2011, *Evolutionary Anthropology*), who proposed the estrus-sex ratio hypothesis. He suggested that prolonged maximal swelling phases increase the number of receptive females in bonobo groups compared to chimpanzees, thereby easing male–male competition and potentially confusing paternity. This could represent an indirect evolutionary benefit for females by reducing coercion and increasing mating opportunities for lower-ranking males. However, our current study suggests the opposite pattern: mating and paternity are strongly skewed toward high-ranking males, which challenges the assumptions of the estrus-sex ratio hypothesis. Although our initial draft focused more on testing this idea, it is no longer a central part in the revised manuscript. Due to extensive restructuring of the Introduction, we regret that we could not integrate this discussion directly into the revised text.

88: Considering the genetic similarity: something seems to be missing in this sentence

- Thank you for pointing this out. The original sentence aimed to suggest that, given the genetic similarity between bonobos and chimpanzees, bonobo males would also allocate mating effort to the maximal swelling phase unless that phase had evolved not to indicate ovulation. However, due to major revisions in the Introduction, this sentence has been removed and could not be revised.

106: P1: given the small number of females/community and the long inter-birth interval, how likely is it that 2 or more females will be receptive simultaneously, ie how biologically meaningful is this prediction?

- Thank you for raising this important biological question. As shown in Table A (formerly Table C) in S1 Text (supplemental information), two females in our study group often gave birth within a two-week span (4 out of 9 births), which may suggest some degree of biological synchrony, although the underlying mechanism remains unclear. Additionally, female bonobos typically resume sexual swelling cycles when their infants reach approximately one year of age (Hashimoto & Ryu et al., 2022, *Primates*), increasing the likelihood of overlapping receptivity among females.

The hypothesis and prediction regarding simultaneous receptivity are now integrated into the end of the revised Introduction as part of the broader research aim. Due to structural changes during revision, we were unable to explicitly incorporate this specific comment into the revised text. Nonetheless, we appreciate your insight and recognize its relevance to future studies on reproductive synchrony and social dynamics in bonobos.

116: what is the evidence from other primates?

- In other primates, including chimpanzees, baboons, and macaques, exaggerated sexual swellings function as graded signals that indicate ovulation probability. This comparative evidence was missing in the original draft. Following your recommendation, we have now integrated this point into the revised Introduction (Lines 73–99), improving the logical flow and broader relevance of the manuscript.

119: the notion of the existence of a male mating effort allocation requires the existence of costs (beyond energy and time) of matings for males. These are never discussed, questioning a key aspect of the entire analysis/paper.

- Thank you for highlighting this important conceptual point. We have now addressed this in the revised Discussion (Lines 348–375). While our study does not directly measure energetic or opportunity costs, we acknowledge that allocating mating effort to conceptive periods likely incurs significant costs for males. We suggest that such costs may help explain why males prioritize late-phase cues like detumescence. Your comment has helped us clarify the evolutionary implications of male behavior, and we will consider this further in future studies.

418: sexual interactions were thus not only recorded via focal animal sampling? Does that mean that the analyses of these key events are based on ad lib data (which would be highly problematic because of the inherent bias of that method)

- Thank you for this critical point. We understand the concern regarding potential bias in ad libitum data collection. While sexual interactions were recorded both during focal sampling and ad libitum observations, the first author and trained assistants made significant efforts to record all copulations as many as possible while following the group. For example, when a copulation was observed but the male partner fled immediately afterward, one or two researchers and assistants would track the male until his identity was confirmed. In a separate study (Ryu, 2024, in Bonobos and People at Wamba), the first author tested the reliability of ad libitum copulation data and found a strong correlation with rates derived from focal sampling, suggesting minimal bias. Moreover, in field studies of tropical forest-dwelling primates, visibility constraints often make complete focal sampling impractical. Even focal sampling can introduce bias if not carefully managed – especially in species with fission-fusion dynamics like bonobos. With this approach, we tried to manage the challenges of field conditions while ensuring the collection of reliable behavioral data.

# Response to Reviewer #3 (general comments)

**Your comments**

This paper examines mating behavior in wild bonobos to test whether males respond to variation in female fecundity. A broader goal is to test whether bonobo females show less reliable cues of fecundity than do other primates, contributing to low rates of sexual aggression in this species.

The data and the analyses are straightforward and convincing. Males engage in more intensive following, solicitation, and competition in the period close to ovulation, and with females who have older infants (an indirect indicator of fecundity). This suggests that, like chimpanzees, bonobo males use simple behavioral rules to concentrate their mating effort on females that are likely to be more fecund. However, the data also suggest that ovulatory signals in bonobos are noisy and thus likely difficult for males to track accurately.

- Thank you very much for your detailed and constructive review. We sincerely appreciate your positive evaluation of our data and analyses. Your recognition that the findings are straightforward and convincing is especially encouraging. We are pleased that our results regarding male mating behavior and its alignment with female fertility were clearly conveyed. We also value your thoughtful interpretation that bonobo males, like chimpanzees, appear to use simple behavioral rules to guide their mating efforts, even under noisy ovulatory signaling.

The following are our point-by-point responses to your comments, questions, and suggestions. Your feedback has helped us improve the manuscript substantially, particularly in terms of clarity, comparative context, and overall focus.

# Response to Reviewer #3 (specific comments)

**Your comments**

Although the data are clearly presented, the authors could do more to situate them in the context of previous studies. Although the discussion briefly mentions comparisons with chimpanzee data, these are not presented systematically. It would be nice to see direct and clear comparisons between these data and published chimpanzee data to be convinced that the findings here relate to species differences in aggression. Is it possible to include a table that presents such data? For example, what is the average age of a female's infant when she resumes ovulatory cycling at different sites? This appears to be younger in bonobos, meaning more cycles to conception. Table C has relevant data on differences in length of the maximal swelling phase, and this should be included in the main text, not buried in the supplemental materials. The more comparative data the authors could include to actually test the hypothesis about species differences the better.

- Thank you for this insightful suggestion. We agree that a more systematic comparison with chimpanzee data would strengthen the manuscript and help clarify species-level differences in reproductive signaling and male behavior. Several comparative studies have already addressed these points (e.g., Deschner & Boesch, 2007, *Int. J. Primatol*.; Wrangham, 2002 and Furuichi & Hashimoto, 2002 in *Behavioural Diversity in Chimpanzees and Bonobos*), and we revisited these sources to confirm that the relevant data are well summarized therein. Unfortunately, we did not find additional recent datasets that would allow us to expand the comparison further, so we were unable to add a new table on infant age at cycle resumption across sites.
- However, following your recommendation, we have moved Table C (now Table 3) from the supplementary materials into the main text. This table presents comparative data on the length of maximal swelling phases across bonobo and chimpanzee populations, which directly supports our discussion on ovulation predictability and mating strategies. We also added clarifying text in the discussion to highlight species differences in infant age at cycle resumption and their implications for mating effort and male aggression. We appreciate your suggestion, which has significantly improved the manuscript’s accessibility and comparative value.

The manuscript is frustrating to read, partly owing to the heavy use of unnecessary acronyms. FMS, MSP, and IFB are all used ad nauseum, forcing the reader to continually remind herself what they mean. I don't see the need for any of these acronyms, but I would pick one maximum to keep. There is no reason not to write these phrases out, or to substitute simpler phrases like "maximal swelling" or "intense following." I can't imagine many readers reading this full paper in its current version.

- Thank you for pointing this out. We fully agree that excessive use of acronyms can hinder readability. In response, we have removed the acronyms FMS, MSP, and IFB throughout the main text. To maintain clarity and conciseness, we retained these abbreviations only in figure and table captions, where space is limited and their use improves visual presentation.

Where appropriate, we replaced the acronyms with simpler and more intuitive phrases such as “maximal swelling” and “intensive following.” Although “intense following” is shorter, we chose to retain “intensive” for consistency with terminology used in the first author’s PhD dissertation. These revisions have improved the clarity and flow of the manuscript, and we appreciate your guidance on this matter.

I don't see the need for Figure 4c, which is almost impossible to interpret, given the 3 dimensions and the opaque shading. The other figures present these data much more clearly.

- Thank you for your feedback regarding Figure 4c. We initially included the 3D plot to illustrate the interaction between the number of males and females with maximal swelling on male aggression rates, and we provided an interactive version via Figshare for better visualization. However, we understand that the static version in the manuscript may be difficult to interpret due to the shading and dimensionality. Following your suggestion, we have revised the figure to use transparent shading and added clearer annotations to improve interpretability. We hope this updated version better communicates the interaction effect. Please let us know if further revision would be helpful.

The discussion could also be trimmed, as it frequently strays into speculation that is not directly supported by the data in the paper. For example, in lines 302-303 the authors state: "If their sperm is limited, they would allocate it more prudently. However, if ejaculations involved in copulation can help with refreshing sperm quality, their behavior is reasonable." This is not helpful because males might be allocating sperm prudently (i.e. investing larger ejaculates in more fecund females) - there are simply no data in this paper to test that one way or the other. If the authors want to make a case that males are mating at rates that would result in sperm depletion, they should do so with data.

- Thank you for your careful reading of the discussion and for highlighting the need to avoid speculative statements not directly supported by our data. In response, we have removed the sentence regarding sperm allocation and other speculative passages that lacked empirical grounding. This revision helped us reduce the discussion by approximately 1,000 words, allowing us to focus more tightly on the core findings and their implications.

In addition to trimming speculative content, we expanded the discussion to place our findings within a broader evolutionary framework. Specifically, we addressed how conspicuous but noisy ovulatory signals in female mammals, particularly bonobos, can persist through male behavioral strategies. We hope the revised discussion is now more concise, empirically grounded, and better aligned with the scope of our data.

# Response to ACADEMIC EDITOR

ADVICE FROM ACADEMIC EDITOR [lightly edited]:

I agree that the work could and should be put into a larger context beyond Bonobos and Chimps, connecting to broader issues of mating tactics and sexual selection. R1 suggests connecting with other species who have anogenital swellings. I agree that discussion should be expanded, but it seems like the minimal amount of broadening. I'd encourage the authors to consider starting even broader, by connecting to the work on male mating tactics and female signals beyond primates. As is, the first paragraph of the paper briefly broadens the scope a bit, but they address the issue more narrowly in the discussion.

- Thank you very much for your thoughtful and constructive advice. In response, we revised both the Introduction and the Discussion to better situate our findings within a broader evolutionary context. The Introduction now starts with general principles of sexual selection and the characteristics of female signaling in mammals, emphasizing that conspicuous signals are relatively rare in females due to their high reproductive investment. We then narrow the focus to exaggerated sexual swellings in primates, framing bonobos within this broader signaling landscape.

In the Discussion, we expanded the scope beyond apes to consider how males across mammalian species respond to a range of ovulatory signals—from brief and reliable cues in species with estrous cycles to more probabilistic and prolonged signals in anthropoid primates with menstrual cycles. We emphasized that bonobo males manage signal uncertainty by concentrating mating effort near detumescence, a terminal cue that reliably marks the end of the fertile window. This post-hoc cue allows males to extract usable timing from imprecise signals, reducing the need for females to produce highly precise ovulatory cues. Such cue integration may help stabilize noisy signaling systems and explain how conspicuous but probabilistic female signals can persist evolutionarily by shifting the cost of signal interpretation onto males.

We hope these revisions address your scope concerns and align the manuscript more closely with the journal’s expectations for broader biological relevance.
