# Supplementary material for: Male bonobo mating strategies target female fertile windows despite noisy ovulatory signals during sexual swelling
Source: PLoS Biol. 2025 Dec 9;23(12):e3003503. doi: 10.1371/journal.pbio.3003503 (PMC12688130; doi:10.1371/journal.pbio.3003503)
Supplement: Attachment — Submitted filename: 1. Response to editorial.docx [file pbio.3003503.s012.docx]

Dear Dr Ryu,

Thank you for your patience while we considered your revised manuscript "Sexual swelling in bonobos: variations, ovulation predictability, and male responses" for publication as a Research Article at PLOS Biology. This revised version of your manuscript has been evaluated by the PLOS Biology editors and the Academic Editor.

Based our Academic Editor's assessment of your revision, we are likely to accept this manuscript for publication, provided you address some remaining points. The Academic Editor has provided detailed comments, which you will find below my signature -- please address them. Please also make sure to address the following data and other policy-related requests.

IMPORTANT: Please ensure that your next revision addresses the following points, as failing to do so may delay publication of your paper:

----------

Dear Dr. Taylor Hart,

Thank you for your careful handling of our revision and for the clear list of remaining items. We appreciate the positive assessment and are pleased to submit the next revision addressing all editorial and policy requests, as well as the Academic Editor’s comments. We respond directly to your comments as follows in the attached document. We appreciate your help with the manuscript, and we hope this revision will fulfill your expectations and the journal’s requirements.

With thanks and best regards,

Heungjin.

Below, we provide our detailed responses to each point raised in your request.

**Title:

We suggest modifying the title of your paper to better emphasize the main findings and fit with our stylistic preferences. Does the following alternative formulation work for you?

"Male bonobo mating strategies target female fertile windows despite noisy ovulatory signals during sexual swelling"

- Thank you for your suggestion. We agree with the new title you suggested. The suggested title is concise and reflects what we report in this study precisely.

**Financial disclosure statement:

Please confirm that grant numbers are included for all funding sources in you Financial Disclosure statement in the manuscript details.

- Confirmed. We have added “The funders had no role in study design, data collection and analysis, decision to publish, or preparation of the manuscript” at the end.

**Ethics:

-- Please include the specific national or international regulations/guidelines to which your animal care and use protocol adhered. Please note that institutional or accreditation organization guidelines (such as AAALAC) do not meet this requirement.

- We have revised our ethics statement as follows by adding international guidelines from the International Primatological Society:

We conducted the study in accordance with the Guidelines for Field Research of Non-Human Primates (Primate Research Institute, Kyoto University, Japan) and the Code of Best Practices for Field Primatology (International Primatological Society).

**Data:

We have a few requests regarding your figures and data availability.

-- We see that you wrote that the data will be made available after acceptance. Please upload the data and include a link so that we can view them now.

- We have uploaded all data and linked them to the corresponding figures in the main manuscript and supplementary figures you suggested. We have reserved permanent DOIs and also added them in the figure and table legends. As the reserved DOIs are not accessible at the moment, we have also included temporary links (private links) for your access to the data and figures. We will publish this data after final acceptance to activate the reserved DOIs.

Note: The sentence about temporary links should be removed before publication.

-- Please also ensure that your uploaded data include the numerical values either in a supplementary excel file or as a permanent DOI’d deposition for the following figures:

1ABCDE

2ABCD

4AB

S1 Fig. A abc

- As we responded previously, we have uploaded all matched data to the Figshare repository and made them available online with temporary and permanent DOIs.

-- Please cite the location of the data clearly in all relevant main and supplementary Figure legends, e.g. “The data underlying this Figure can be found in S1 Data” or “The data underlying this Figure can be found in https://doi.org/10.5281/zenodo.XXXXX”

- Following your guidance, we have included data links (DOI links) in the figure legends as well as in the Data and Materials Availability section, as follows:

“All data for statistical models and figures, including R code, are available in the Figshare repository (private link until publication: https://figshare.com/s/e15a4478183a1a9008ac, reserved DOI for publication: https://doi.org/10.6084/m9.figshare.30403564).”

Note: The part about “private link until publication” and “reserved DOI for publication” must be removed before publication.

-- We suggest some modifications of the naming scheme for your supplementary figures: instead of Fig. A, containing panels a, b, c, can this be changed to Fig. S1 containing panels A, B, C? This will make your paper more consistent with others at PLOS Biology and help avoid confusion among readers.

- Based on our communications on the issues via email, we have changed the numbers and names of the supplementary figures following your suggestion. Fig A in S1 Text is now S1 Fig, and Fig B in S1 Text is now S2 Fig, and so on. They have also been separately uploaded to the Figshare repository.

-- Supplementary files (e.g., excel). Please ensure that all data files are uploaded as 'Supporting Information' and are invariably referred to (in the manuscript, figure legends, and the Description field when uploading your files) using the following format verbatim: S1 Data, S2 Data, etc. Multiple panels of a single or even several figures can be included as multiple sheets in one excel file that is saved using exactly the following convention: S1_Data.xlsx (using an underscore).

- We uploaded all files, using mostly CSV format, except for the data for S4 Fig (in XLSX format), to the Figshare data repository along with the R code. They can be downloaded at once and directly imported into R for replication of data analysis and figure creation.

----------

As you address these items, please take this last chance to review your reference list to ensure that it is complete and correct. If you have cited papers that have been retracted, please include the rationale for doing so in the manuscript text, or remove these references and replace them with relevant current references. Any changes to the reference list should be mentioned in the cover letter that accompanies your revised manuscript.

- We have checked the listed references and confirmed that they contain no errors.

In addition to these revisions, you may need to complete some formatting changes, which you will receive in a follow up email. A member of our team will be in touch with a set of requests shortly. If you do not receive a separate email within a few days, please assume that checks have been completed, and no additional changes are required.

We expect to receive your revised manuscript within two weeks.

To submit your revision, please go to https://www.editorialmanager.com/pbiology/ and log in as an Author. Click the link labelled 'Submissions Needing Revision' to find your submission record. Your revised submission must include the following:

- a cover letter that should detail your responses to any editorial requests, if applicable, and whether changes have been made to the reference list

- a Response to Reviewers file that provides a detailed response to the reviewers' comments (if applicable, if not applicable please do not delete your existing 'Response to Reviewers' file.)

- a track-changes file indicating any changes that you have made to the manuscript.

NOTE: If Supporting Information files are included with your article, note that these are not copyedited and will be published as they are submitted. Please ensure that these files are legible and of high quality (at least 300 dpi) in an easily accessible file format. For this reason, please be aware that any references listed in an SI file will not be indexed. For more information, see our Supporting Information guidelines:

https://journals.plos.org/plosbiology/s/supporting-information

*Published Peer Review History*

Please note that you may have the opportunity to make the peer review history publicly available. The record will include editor decision letters (with reviews) and your responses to reviewer comments. If eligible, we will contact you to opt in or out. Please see here for more details:

https://plos.org/published-peer-review-history/

- We agree to the publication of the peer review history.

*Press*

Should you, your institution's press office or the journal office choose to press release your paper, please ensure you have opted out of Early Article Posting on the submission form. We ask that you notify us as soon as possible if you or your institution is planning to press release the article.

- Unfortunately, there are no press release plans from my university.

*Protocols deposition*

To enhance the reproducibility of your results, we recommend that if applicable you deposit your laboratory protocols in protocols.io, where a protocol can be assigned its own identifier (DOI) such that it can be cited independently in the future. Additionally, PLOS ONE offers an option for publishing peer-reviewed Lab Protocol articles, which describe protocols hosted on protocols.io. Read more information on sharing protocols at https://plos.org/protocols?utm_medium=editorial-email&utm_source=authorletters&utm_campaign=protocols

Please do not hesitate to contact me should you have any questions.

Sincerely,

Taylor

Taylor Hart, PhD,

Associate Editor

thart@plos.org

PLOS Biology

Editorial Comments from the Academic Editor:

- Thank you for your critical and constructive guidance, which has greatly improved the manuscript. In particular, your suggestion on the precise use of “signal” and “cue” was very helpful. The following are our responses and revisions based on your suggestions. We hope that our revision meets your expectations.

Abstract:

Line 17-18: I suggest “This raises the question about how males successfully time mating, particularly when ovulation is difficult to predict from such signals. To address this question in bonobos, we combined…” Note that I changed “cue” to “signal”, which is the consistent with how the terms are currently used in the animal communication literature (a cue provides information incidentally, whereas a signal has been naturally selected to convey information that elicits a response from the receiver). I suggest doing so throughout the paper.

- Thank you very much for your suggestions. We acknowledge that our use of the terms "cues" and "signals" was inconsistent. Following your recommendations, we have revised the manuscript to use "signals" throughout, including in the abstract (see also L80, L123). We retained the term "cues" only in reference to detumescence as a post-ovulatory cue, as it is difficult to definitively categorize detumescence as a signal based on current knowledge. Additionally, in the previous version, we used both "post-hoc cues" and "post-ovulatory cues" to describe detumescence as a late-phase indicator. For consistency and clarity, we have replaced all instances of "post-hoc cues" with "post-ovulatory cues.

Line 20: define detumescence in a few words as part of the sentence. It’s a word most readers will need to Google. For example, “By estimating day-specific ovulation probabilities relative to the onset of the maximal swelling and subsidence of swelling (detumescence), we…”

- We revised it following your suggestions (LL 20-22).

From

By estimating day-specific ovulation probabilities relative to the onset of the maximal swelling phase and detumescence, we also examined how male efforts correlate with female fertility.

To

By estimating day-specific ovulation probabilities relative to the onset and subsidence (detumescence) of maximal swelling, we also examined how male efforts correlate with female fertility.

Introduction:

I appreciate that the authors broadened the introduction further beyond bonobos. It might be a little too broad in some parts, however, as it takes a little too long to reach the key set-up for your study. I made a few suggestions for making these same points in a more succinct way.

- Thank you for your thorough review and thoughtful suggestions. They significantly improved our manuscript and gave us a valuable opportunity to reframe our findings from a broader evolutionary perspective on female reproductive signaling in mammals. We mostly revised our introduction in response to your suggestions and comments. We also slightly shortened the introduction by removing less relevant parts – specifically, details about signaling systems in mammals. We hope that our revision meets your expectations

Line 41: “…success often facilitated…” Or just drop the first sentence and begin with “Darwin’s theory of sexual selection explains how traits.”

- We removed the first sentence and started the first sentence as you suggested (LL 41-42.)

From

Sexual selection operates when individuals differ in their ability to compete for accessing mates and fertilizations, resulting in differences in reproductive success facilitated by mate choices in the opposite sex [1,2]. Since Darwin’s introduction of sexual selection [3], it has successfully explained how traits that appear costly for survival can evolve in one sex, if they give advantages in mate competition or attraction [4,5].

To

Darwin’s theory of sexual selection [1] explains how traits that appear costly for survival can evolve in one sex if they give advantages in mate competition or attraction [2,3].

Lines 44-45: Some great reviews have shown that these patterns are common, but also highlight the wide diversity of sex roles. Maybe adjust phrasing to reflect this. “Males are the sex that more often develops conspicuous signals of competitive ability, and females are often choosier about their mates [1,2].” Possible citations: https://www.science.org/doi/10.1126/sciadv.1500983 https://journals.plos.org/plosbiology/article?id=10.1371/journal.pbio.3001916

- We revised it following your suggestion. We also added references you suggested (LL 42-44).

From

Under this scenario, males are typically the sex that develop conspicuous traits signaling their competitive quality, and females are usually the sex that choose [1,2].

To

Under this scenario, males are typically the sex that more frequently develops conspicuous signals of competitive ability, and females are often more selective about their mates [4–7].

Line 52-54: I don’t think the *Mus* example is needed.

- Following your suggestion, we removed the *Mus* example (L 51).

Lines 55-57: It’s worth mentioning in this paragraph the possibility that females signal to manipulate male-male competition--either to reduce it, affect its timing, or to incite competition. In any of these cases, it’s a means of females indirectly choosing their mates (https://doi.org/10.1111/j.1558-5646.1996.tb03911.x ). Some parts of this are discussed later, but seems worth raising here, where you talk about the different reasons females may signal during reproduction.

- We were previously unaware of the concept of indirect female choice. We now realize that the role of female signals in mammals can be well understood within the framework of indirect female choice. We have included your suggestion in LL 47–50 and 53–56 (Red: newly added).

LL 47-50

Female signaling has therefore been viewed mainly as a means of advertising fertility or reproductive condition over short time windows that could influence male-male competition – either by reducing it, affecting its timing, or inciting it – rather than as prolonged displays of competitive ability [5,13,14].

LL 53-56.

Nevertheless, even in these cases, females typically compete for reproductive opportunities or for raising offspring, rather than for direct access to mates [15,17], suggesting a major role of female conspicuous signaling can be explained from the perspective of indirect female choice [18].

Lines 72-73: The names of these hypotheses are confusing, though I realize that they are used commonly in the literature. The problem is that a “reliable indicator” could reliably indicate ovulation, and quality indicators are typically graded signals, so the reader needs to remember which is which. I would consider calling them the “quality indicator” (or “quality signal”) and the “ovulation signal” hypothesis. Those terms distinguish them by what the signal is indicating. You could do that by saying “The best-known hypotheses are the quality indicator hypothesis (often called reliable indicator, [27]) and the ovulation signal hypothesis (often called “graded signal”, [34]).” Or something along those lines. This is only a suggestion and up to the authors.

- Thank you for your suggestions. We agree with your points. The terminology is indeed confusing, particularly the "reliable indicator hypothesis." If a signal has evolved within a communication system, it is typically reliable over evolutionary timescales; otherwise, the system would likely collapse. We were initially concerned because these two terms are commonly used in the literature on sexual swelling in primates, and changing them might cause confusion. However, considering the broader readership of PLOS Biology, we decided to follow your suggestions. We replaced "reliable indicator hypothesis" with "quality indicator hypothesis" and "graded signal hypothesis" with "graded ovulatory signal hypothesis" throughout the manuscript. It was difficult to replace "graded signal hypothesis" with "ovulation signal hypothesis," as the former is well-established and widely documented across primate species. Therefore, we used "graded ovulatory signal hypothesis."

Line 75: It’s not clear to me why female signaling creates an arena for female-female competition. I would view the sexual signals as a consequence of the opportunity for competition. I suggest dropping the last clause, after “sexual swelling”

- Thank you for your suggestion. You are right that sexual swelling signals are a consequence rather than a driver of female–female competition. What we intended to convey is that the evolutionary emergence of sexual swelling may reflect the presence of female–female competition over access to males. The previous sentence did not accurately express this idea. We attempted to remove the latter part following your suggestion. However, because the assumed causation of the evolutionary rise of sexual swelling in the quality indicator (formerly reliable indicator) hypothesis is female-female competition for expressing reproductive quality, we revised the sentence as follows (LL 72–75).

From

The reliable indicator hypothesis proposes that variation in swelling size between females reflects differences in reproductive quality, leading males to preferentially invest in females with larger swellings and thereby creating an arena for female-female competition for access to males [34,35].

To

The female quality indicator hypothesis proposes that variation in swelling size between females reflects differences in reproductive quality, leading males to preferentially invest in females with larger swellings, and is therefore assumed to be shaped by female-female competition for access to males [36,37].

Line 88-90: I suggest “Despite this progress, further investigation is necessary to better understand the relative importance of mate choice and male coercion in determining fertilization, and how the conspicuity and reliability of female signals influences mating and social behaviors.”

- We revised the part following you suggestions (LL 88–90).

Lines 90-92: Do you mean these are consequences of the evolution of increased conspicuity and reduced reliability? The wording implies that someone (the female or the researcher) is actively able to control the signal.

- Thank you for pointing this out. As you pointed out, our previous sentence implies agency that holds the control, rather than implying natural or sexual selection that works behind. To avoid any suggestion of agency, we revised the sentence as follows (LL90–92).

From

Increasing signal conspicuity likely carries physiological costs that could otherwise be allocated to reproduction, and lowering reliability by allowing ovulation outside the signal risks introducing errors even for female choice [23,29].

To

Selection favoring greater signal conspicuity likely carries physiological costs that could otherwise be allocated to reproduction, and weaker coupling between the signal and the timing of ovulation increases the risk of mating-decision errors even for females [25,31].

Lines 101: The paragraph above this one is a nice set up for what you plan to study here. This might therefore be a good place to shift into the “In this study…” part of the introduction. I suggest simply moving the sentence currently on line 120 (“In this study, we tested the signal reliability of the sexual swelling as a graded ovulatory signal in a wild bonobo group.”) up to the beginning of this paragraph. Replace the sentence in line 120 with something like “To examine the signal reliability of sexual swellings as a graded ovulatory signal, we first investigated…”

- Thank you very much for your suggestions. We revised the manuscript accordingly, which greatly improved its flow. Please see LL 101–102 and L 123.

Line 109: Why is this called the “estrus-sex-ratio hypothesis”? I assume it increases the number of estrus females per male. This hypothesis isn’t mentioned again until the results, so the predictions aren’t made clear.

- Thank you for your comments. You are right about this point. As you noted, it increases the number of estrous females per male. We revised the sentence to define the term and state the prediction you mentioned more explicitly (LL 108– 111). By doing so, our interpretation of the result in LL 223–225, which aligns with the estrus sex ratio, has become clearer and easier to understand. The revised sentence is as follows.

From

Frequent swelling appearance in bonobos may lengthen receptive periods and diffuse male competition by increasing opportunities to mate with females with maximal swelling – the estrus-sex-ratio hypothesis [50,55].

To

The estrus-sex-ratio hypothesis predicts that frequent swelling lengthens receptive periods and increases the number of receptive (estrous) females per male, thereby reducing male-male competition and increasing mating opportunities, including for low-ranking males, in bonobos [52,57].

Lines 116-117: Is there evidence to support that this is a highly costly signal? I suggest “potentially costly”. The latter half of this sentence implies that sperm competition is bad for the female. Do you mean that the signal might increase male coercion? Also, the signal might hinder the female’s ability to choose her mate, but presumably not the preference itself.

- Thank you for your suggestions and questions. As you pointed out, the costs associated with prolonged swelling are mostly assumed rather than quantitatively tested. Following your comments, we revised “costly” to “potentially costly” and adjusted other parts to integrate your suggestions. We did not expect that the signal could increase male coercion, particularly in bonobos. Our point was that a prolonged signal could potentially increase randomness in female mate choice, as a higher number of mating partners may lead to increased sperm competition. This, in turn, could influence the randomness of her choice, unless her choice is biased toward males with better sperm quality, which we do not rule out. The revised sentence is as follows (LL 117 – 119).

From

It also raises the question of why females maintain such a costly and prolonged ovulatory signal that could elevate sperm competition and potentially hinder preferences for high-ranking males [63].

To

It also raises the question of why females maintain such a potentially costly and prolonged ovulatory signal that may hinder females’ ability to choose high-ranking males, given that prolonged receptive periods can increase sperm competition [65].
